# SM2RAIN-Climate, a monthly global long-term rainfall dataset for climatological studies

**DOI:** 10.1038/s41597-023-02654-6

**Published:** 2023-10-31

**Authors:** Hamidreza Mosaffa, Paolo Filippucci, Christian Massari, Luca Ciabatta, Luca Brocca

**Affiliations:** Research Institute for Geo-Hydrological Protection, National Research Council, Perugia, Italy

**Keywords:** Climate sciences, Hydrology

## Abstract

A reliable and accurate long-term rainfall dataset is an indispensable resource for climatological studies and crucial for application in water resource management, agriculture, and hydrology. SM2RAIN (Soil Moisture to Rain) derived datasets stand out as a unique and wholly independent global product that estimates rainfall from satellite soil moisture observations. Previous studies have demonstrated the SM2RAIN products’ high potential in estimating rainfall around the world. This manuscript describes the SM2RAIN-Climate rainfall product, which uses the European Space Agency (ESA) Climate Change Initiative (CCI) soil moisture v06.1 to provide monthly global rainfall for the 24-year period 1998–2021 at 1-degree spatial resolution. The assessment of the proposed rainfall dataset against different existing state-of-the-art rainfall products exhibits the robust performance of SM2RAIN-Climate in most regions of the world. This performance is indicated by correlation coefficients between SM2RAIN-Climate and state-of-the-art products, consistently exceeding 0.8. Moreover, evaluation results indicate the potential of SM2RAIN-Climate as an independent rainfall product from other satellite rainfall products in capturing the pattern of global rainfall trend.

## Background & Summary

Rainfall is a vital component of the hydrological water cycle, which controls the balance of water and energy on the Earth. As the most Essential Climate Variable by the Global Climate Observing System (GCOS)^1^, accurate, long-term, and consistent rainfall datasets are crucial in many applications, including water resource management^2^, climate studies^3^, and drought monitoring^4^. Ground-based measurements (rain gauges and radars), reanalysis-based, and satellite-based observations are the most important approaches for rainfall estimation. Rain gauges are considered as the most reliable rainfall data sources, but they have two main limitations: (1) low-density spatial coverage in many regions of the world and (2) limited representative area around the gauge^5^. Meteorological radar estimates rainfall with high spatial resolution. However, it suffers from high-cost maintenance and a lack of accessibility limitations^6^. Reanalysis products, generated through numerical models and data assimilation algorithms, introduce uncertainties^7^. Satellite-based rainfall products, with their high spatial and temporal resolution, offer an alternative rainfall estimation technique. Notably, various precipitation databases have been developed, including those based on station observations (e.g., Global Historical Climatology Network(GHCN)^8^, Climate Prediction Center (CPC), Global Precipitation Climatology Centre (GPCC)^9^, Climate Research Unit (CRU)^10^), radars (e.g., Stage-IV^11^, multi-Radar/Multi-Sensor (MRMS)^12^ and NIMROD^13^), reanalysis (e.g., Research and Applications (MERRA)^14^, the Japanese 55-year Reanalysis (JRA55)^15^, and the European Centre for Medium-Range Weather Forecasts (ECMWF)^16^ Re-Analysis (ERA)), satellite-derived data (e.g., SM2RAIN^17^, the Integrated Multi-satellitE Retrievals for GPM (IMERG)^18^), and combinations of multiple resources (e.g., Multi-Source Weighted-Ensemble Precipitation (MSWEP)^19^, Frequent Rainfall Observations on GridS (FROGS)^20^). These precipitation databases provide valuable information in its spatio-temporal scale, although they are also subject to uncertainties, for example the multi-platform satellite precipitation products can also be impacted due to changes in the satellite constellation over time, which can be crucial for climate studies^21,22^.

There are two main approaches, including top-down and bottom-up, for rainfall estimation from satellite observations^17^. In the top-down approach, rainfall is obtained from the combination of the Geostationary (GEO) and Low Earth Orbiting (LEO) satellites sensors whose rainfall is related to the emitted or reflected radiation from clouds or rain droplets^23^. In the bottom-up approach, rainfall is estimated from soil moisture (SM) observations through the SM2RAIN algorithm^17^. In this algorithm, soil is considered as a natural rain gauge and the algorithm is based on the inversion of the soil water balance equation. The main difference between these two approaches algorithms is that top-down approaches are based on instantaneous measurements of rainfall which may cause the underestimation of rainfall when the satellites do not pass over the rainfall event. Differently, bottom-up approaches provide accumulated rainfall between two consecutive SM measurements.

The bottom-up approach has been applied at a global scale^23,24^ and a local scale in Asia^25–28^, Australia^29,30^, Europe^31,32^, Africa^33,34^, and America^35,36^. More than 150 papers have been published in peer-reviewed journals related to SM2RAIN rainfall products, which have highlighted the high potential of this approach over different parts of the globe and also the effectiveness of them in many applications including flood prediction^37^, water resources management^38^, and prediction of landslide^39^, soil erosion^33^, and crop yield^40^.

In the last eight years, several SM2RAIN rainfall products were developed by forcing different SM satellite observations into the algorithm, including SMOS^41^ (Soil Moisture Ocean Salinity mission), ASCAT^24^ (Advanced SCATterometer), AMSR2^42^ (Advanced Microwave Scanning Radiometer 2), Sentinel-1^43^, ESA CCI SM^44^ (the European Space Agency Climate Change Initiative), and SMAP^45^ (Soil Moisture Active and Passive). These studies demonstrate that the accuracy of the SM2RAIN rainfall products is highly dependent on the accuracy of satellite soil moisture observations. However, it is important to note that the accuracy of these products is not uniform over the globe. The precision of the retrieved soil moisture data, and thus the resultant precipitation estimates, can vary due to a range of factors. These factors include the accuracy of the retrieval algorithm, the sampling interval, and the number of retrievals possible in remote areas for each sensor. In general, low performance of soil moisture products over densely vegetated areas, frozen soils, snow-covered soils, and complex topography regions are expected.

Long-term rainfall datasets are fundamental for climatological studies and analyses of spatiotemporal rainfall variations. However, existing SM2RAIN rainfall products do not offer extensive records. To address this gap, we present a long-term, gridded rainfall dataset that opens up potential for various applications. This dataset provides essential data for understanding global patterns and trends, aiding climate change research, water resource management strategies, hydrological modelling, and large-scale hydro-ecological studies. Therefore, the goal of this study is to build the global SM2RAIN-Climate rainfall product with monthly temporal and 1° spatial resolutions for the 24-year period 1998–2021. This product presents a distinct and independent addition to existing rainfall datasets for climatological studies and is based on the ESA CCI SM products^16^.

## Methods

This study utilizes a variety of datasets to achieve its objectives. The ESA CCI soil moisture dataset serves as the foundation for SM2RAIN-Climate generation. For validation purposes, we employ three distinct rainfall datasets representing gauge-based, reanalysis, and satellite-based products: Global Precipitation Climatology Centre (GPCC), European ReAnalysis 5th Generation (ERA5)-Land, and Climate Hazards Group InfraRed Precipitation with Station data (CHIRPS), respectively.

### ESA CCI soil moisture v06.1

In 2012, ESA developed the first long-term global satellite-based SM datasets as a part of the Climate Change Initiative program. The dataset contains three products, including active, passive, and combined at 0.25° spatial and daily temporal resolution. The active product is built by merging scatterometer soil moisture data for the period of 1991–2021. Passive product retrievals from multiple passive microwave sensors and the data available for 1978–2021. The ESA CCI combined product was developed by merging the active and passive sensors. The active and passive ESA CCI SM v06.1^16^ data are used in this study. The products are accessible through https://www.esa-soilmoisture-cci.org/. Table 1 summarizes the sensors used for the active and passive products of ESA CCI SM.

**Table 1:** Overview of the sensors used for active and passive products of ESA CCI SM^16^.

Active Sensor	Temporal interval
**AWI-WS**	Aug 1991–Dec 2006
**ASCAT-A**	Jan 2007–Dec 2020
**ASCAT-B**	Nov 2012–Dec 2021
**ASCAT-C**	Nov 2018–Dec 2021
**Passive Sensor**	**Temporal interval**
**SMMR**	Nov 1978–Aug 1987
**SSM/I**	Sep 1987–Jun 2002
**TMI**	Jan 1998–Dec 2013
**AMSR-E**	Jul 2002–Oct 2011
**WindSat**	Oct 2007–Jun 2012
**SMOS**	Jan 2018–Dec 2021
**FY-3B**	Jun 2011–Aug 2019
**FY-3C**	Sep 2013–Aug 2021
**FY-3D**	Jan 2019–Aug 2021
**AMSR2**	Jul 2012–Dec 2021
**GPM**	Mar 2014–Dec 2021
**SMAP**	Apr 2015–Dec 2021

### Global precipitation climatology centre (GPCC) v2020

This product was developed in 1989 at the National Meteorological Service of Germany as a contribution to the World Climate Research Programme (WCRP). This product provides the global daily rainfall dataset at 1° spatial resolutions for the period after 1982. This dataset is based on *in situ* rain gauge data which contain the information of +84000 stations. The description of the GPCC dataset^9^ can be found in Schneider *et al*.^46^ The dataset is available on the website https://opendata.dwd.de/climate_environment/GPCC/html/download_gate.html.

### European ReAnalysis 5th Generation (ERA5)-Land

ERA5-Land is the land component of the ERA5 reanalysis dataset that is produced by the European Centre for Medium-Range Weather Forecasts (ECMWF)^47^. The dataset is available with 0.1° spatial and hourly temporal resolution from 1950 to the present. In this study, three components, including soil temperature for the layer of 0–7 cm, snowfall, and precipitation, are used by regridding to 1° spatial resolutions and averaged to daily temporal resolution. Also, by eliminating the snowfall component of ERA5 from the ERA5-Land total precipitation, daily rainfall data were derived, which is used in the technical validation of SM2RAIN-Climate. The ERA5 dataset is available via the Copernicus Climate Data Store (CDS) (https://cds.climate.copernicus.eu/.

### Climate hazards group infrared precipitation with station data (CHIRPS) v2.0

The United State Geological Survey (USGS), and Climate Hazard Center of the University of California-Santa Barbara developed the CHIRPS dataset, which is a rainfall dataset with land-only quasi-global coverage (50°S–50°N) at 0.05 spatial and daily temporal resolutions for the period from 1981 to present. This product consists of both satellite and gauge observation information. The details of CHIRPS can be found in Funk *et al*.^48^ and the data can be downloaded at https://www.chc.ucsb.edu/data/chirps. We used this dataset in the technical validation section by regridding CHIRPS to 1° spatial resolutions by using the bilinear interpolation method.

#### SM2RAIN-climate generation

**Fig. 1:**
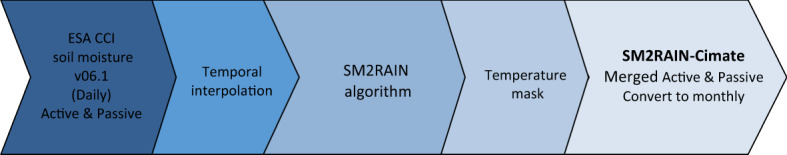
The methodological framework used to generate the SM2RAIN-Climate rainfall product.

The SM2RAIN-Climate rainfall product is developed by using the ESA CCI SM product as input into the SM2RAIN algorithm. Specifically, three steps are implemented, including pre-processing of ESA CCI SM, applying SM into the SM2RAIN algorithm, and rainfall post-processing (Fig. 1). Due to the low temporal frequency of ESA CCI SM before 1998^49^, all processes are applied for the period 1998–2021. As shown in Table 1, the ESA CCI SM products are built by different sensors during the time, and therefore, the dataset generation is done separately in different periods. These periods, specifically 1998–2001, 2002–2006, 2007–2012, 2013–2014, and 2015–2021, were chosen based on the operational periods of different sensors used in the active and passive products. Each period represents a unique combination of sensors with varying characteristics and sensitivities to soil moisture. Moreover, all the process is done separately for ESA CCI SM active and passive.

The details of the methods are as follows.

### Pre-processing of ESA CCI soil moisture

The pre-processing procedures are performed on the daily active and passive ESA CCI SM products before using them into the SM2RAIN algorithm. SM products are spatially regridded to 1° spatial resolutions using the bilinear interpolation method. Moreover, to fill the temporal gaps of the SM time series, linear interpolation in time was applied on gaps with a duration lower than three days. This process of linear interpolation ensures a more complete and continuous dataset for the application of the SM2RAIN algorithm. After this step, the datasets are ready to be applied into the SM2RAIN algorithm.

### SM2RAIN algorithm description

The SM2RAIN algorithm^17^ was developed to estimate rainfall from two successive SM observations by inverting the soil water balance equation as follows:

1 $${Z}^{* }\frac{dSM\left(t\right)}{dt}=p\left(t\right)-g\left(t\right)-r\left(t\right)-e\left(t\right)$$

Where Z^*^ (mm) is the soil water capacity obtained by soil porosity times soil layer depth, *SM*(*t*) (−) is the relative SM and *p*(*t*), *r*(*t*), *e*(*t*), and *g*(*t*) are the rainfall, surface runoff, evaporation, and drainage rate (mm d^−1^), respectively. Since this equation is used to estimate rainfall during the rainfall event, evaporation and surface runoff are assumed to be negligible^50^. The drainage rate depends on SM, which is obtained from Famiglietti and Wood’s equation^51^. Hence, the rainfall rate can be estimated by rewriting Eq. (1) as follows:

2 $$p(t)={Z}^{* }\frac{dSM(t)}{dt}+a.SM{(t)}^{b}$$

Where a(mm d^−1^) and b(−) are the saturated hydraulic conductivity and the Famiglietti and Wood’s equation exponent, respectively. Before SM data is used in the algorithm, it undergoes another modification, which is an exponential filtering. This filtering step is used to mitigate the effects of temporal noise in the satellite SM observations. We apply the modified exponential filter equation as described by Brocca *et al*.^24^, where the characteristic time length parameter (T) is considered a function of SM through a 2-parameter power law. The two parameters of the modified exponential filter equation, a, b, and Z^*^ are obtained by point-by-point calibration of SM2RAIN algorithm against the GPCC rainfall dataset. For calibration, the minimization of root means square error (RMSE) between the SM2RAIN rainfall estimates and GPCC rainfall is used as an objective function. The calibration is performed separately for each time period of analysis, and for ESA CCI SM active and passive products. Calibrating SM2RAIN separately for each period ensures accuracy and consistency across different time periods. After the calibration, the datasets in the different periods are temporally merged to obtain two rainfall products from active and passive ESA CCI SM, respectively, for the period 1998–2021.

### Post-processing of rainfall

After the estimation of the daily active and passive rainfall through the SM2RAIN algorithm, the soil temperature mask is applied to remove the area with SM retrieval issues (snow covered and frozen soil). For this purpose, a thorough sensitivity analysis was conducted to determine an appropriate criterion. During the sensitivity analysis, different threshold values (0 to 3 °C) for the ERA5-Land soil temperature were evaluated to assess their impact on the post-processing of rainfall. Based on the analysis, the rainfall on days with the ERA5-Land soil temperature below 3 °C is excluded from the time series. Following this step, to gain from the advantages of both active and passive rainfall estimations, they are combined. This combination is achieved by employing a methodology proposed by Kim *et al*.^52^, that maximizes the correlation between estimated rainfall and GPCC. This method is expressed through the equation:

3 $${P}_{SM2RAIN-Climate}=k.{P}_{active}+(1-k).{P}_{passive}$$

Where *P*_*active*_ and *P*_*passive*_ are the active and passive rainfall estimation and *P*_*SM2RAIN-Climate*_ is the merged product. *k* is calculated as follows:

4 $$k=\frac{{\rho }_{AG}-{\rho }_{AG}.{\rho }_{PG}}{{\rho }_{PG}-{\rho }_{AP}.{\rho }_{AG}+{\rho }_{AG}-{\rho }_{AP}.{\rho }_{PG}}$$

In this equation, the subscripts A, P, and G denote the active, passive, and GPCC datasets, respectively, and ρ signifies the Pearson’s correlation coefficient between two datasets. This approach operates under the assumption that both rainfall estimations are unbiased and that there is a correlation between the errors of these estimations and the GPCC. In case one of the active or passive rainfall estimations is not available, the remaining one is incorporated into the merged product. After generating the daily SM2RAIN-Climate product, the product is converted to monthly by scaling the available daily data. In cases where there are missing values, a threshold is defined to identify months with a high percentage of missing data. If the percentage of daily missing data for a particular month exceeds the threshold, that month is considered with no data. However, for months with a lower percentage of missing data, the available daily values are averaged, and the resulting average is then multiplied by the number of days in the month to estimate the accumulated monthly rainfall.

## Data Records

Based on the methodology outlined above, the SM2RAIN-Climate global monthly rainfall dataset is generated in the period 1998–2021. The product is named SM2RAIN-Climate, and it is publicly available at (10.5281/zenodo.7276469)^53^ in NetCDF format. As mentioned above, we have considered the temperature mask in data post-processing and a threshold value (percentage of missing data) to take into account missing data within a month. Specifically, we have considered two threshold values of 66% and 20% to obtain a lower and higher quality product, respectively. The lower quality product (threshold equal to 66%) has better spatial and temporal coverage, and hence depending on the application, the user can select the more suitable product. Therefore, four different SM2RAIN-Climate datasets are provided. For each dataset, the spatial grid (latitude and longitude), the rainfall values, and the mask type is defined in each NetCDF file. The flag value convention and the specifications of each dataset are shown in Table 2. These multiple datasets allow users to choose the most suitable option based on their specific needs and application requirements.

**Table 2:** SM2RAIN-Climate filenames and flag value convention.

# product	File name	Flag_value	Flag_meaning
**1**	SM2RAIN-Climate-monthly-1998–2021-f1	1	Missing threshold 66, without temperature mask
**2**	SM2RAIN-Climate-monthly-1998–2021-f2	2	Missing threshold 20, without temperature mask
**3**	SM2RAIN-Climate-monthly-1998–2021-f4	4	Missing threshold 66, temperature mask
**4**	SM2RAIN-Climate-monthly-1998–2021-f5	5	Missing threshold 20, temperature mask

## Technical Validation

The technical validation of the SM2RAIN-Climate product involves a comparison with various global rainfall products to assess its performance. For this purpose, three distinct datasets, namely GPCC, ERA5, and CHIRPS, have been selected. These datasets represent gauge-based, reanalysis, and satellite-based rainfall products, respectively, and have available data during the period from 1998 to 2021. It’s important to note that the NASA Integrated Multi-satellitE Retrievals for Global Precipitation Measurement (IMERG) dataset is not available for the whole period of 1998–2021 and therefore was not included in the validation process. Four quantitative statistical metrics, including correlation coefficient (CC), root mean square error (RMSE), bias (BIAS), and Kling-Gupta Efficiency (KGE) are calculated for the evaluation of the SM2RAIN-Climate product (note that a positive BIAS represents the overestimation of SM2RAIN-Climate against the other products).

### Global scale assessment

The assessment of SM2RAIN-Climate products against ERA5, GPCC, and CHIRPS are shown in Table 3. Overall, the SM2RAIN-Climate product with a 66 percent threshold and no temperature mask (flag_value=1) demonstrated relatively lower quality. However, the other SM2RAIN-Climate products exhibited slightly better performance compared to ERA5, GPCC, and CHIRPS (flag_value=1), with a 2.62%, 2.41%, and 1.94% improvement in correlation coefficient (CC), respectively, for the higher quality/lower temporal product (flag_value=5). Given that this product (flag_value=1) exhibits the lowest missing values, its validation is presented in this paper.

**Table 3:** Evaluation of different products of SM2RAIN-Climate against other products.

# product	Flag_value	CC	RMSE	BIAS	KGE	% Sample Size Change compared to product #1
**Against ERA5**						
1	1	0.799	52.730	1.040	0.741	0.00
2	2	0.816	51.752	−1.088	0.747	9.97
3	4	0.799	53.717	−0.337	0.746	6.09
4	5	0.820	52.446	−2.265	0.748	16.78
**Against CHIRPS**						
1	1	0.824	53.286	3.170	0.759	0.00
2	2	0.837	52.106	2.237	0.765	7.78
3	4	0.820	54.459	3.084	0.762	3.83
4	5	0.840	52.353	1.849	0.768	12.52
**Against GPCC**						
1	1	0.829	49.777	3.162	0.750	0.00
2	2	0.846	48.662	1.746	0.756	9.97
3	4	0.828	50.987	2.574	0.755	6.09
4	5	0.849	49.365	1.073	0.759	16.78

**Fig. 2:**
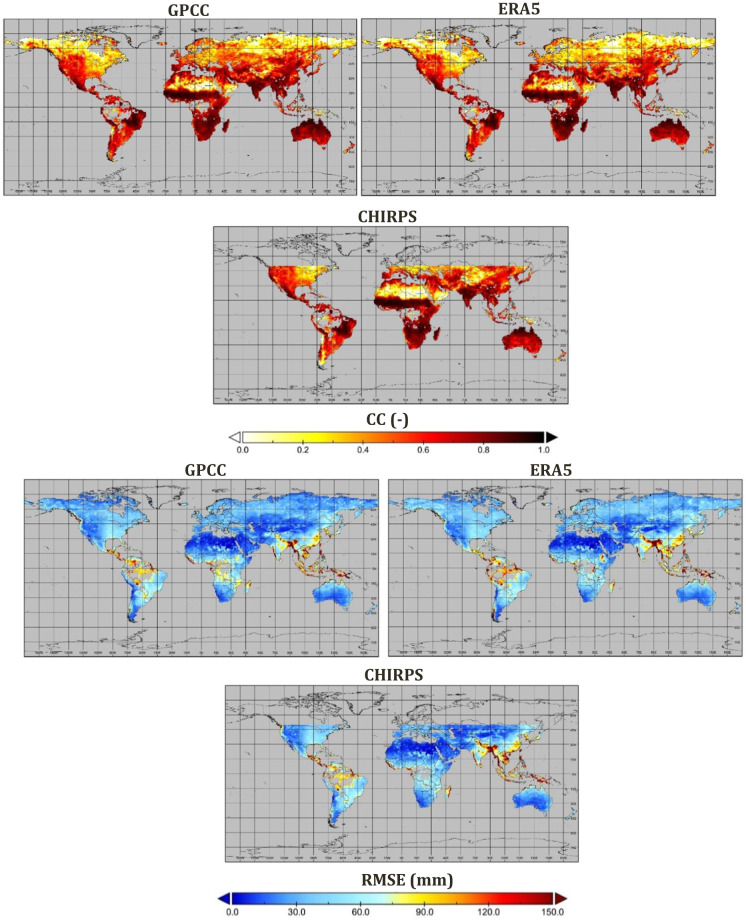
Spatial evaluation of SM2RAIN-Climate (product #1 in Table 2) dataset against GPCC, ERA5, and CHIRPS in terms of correlation coefficient (CC) and root mean square error (RMSE).

**Fig. 3:**
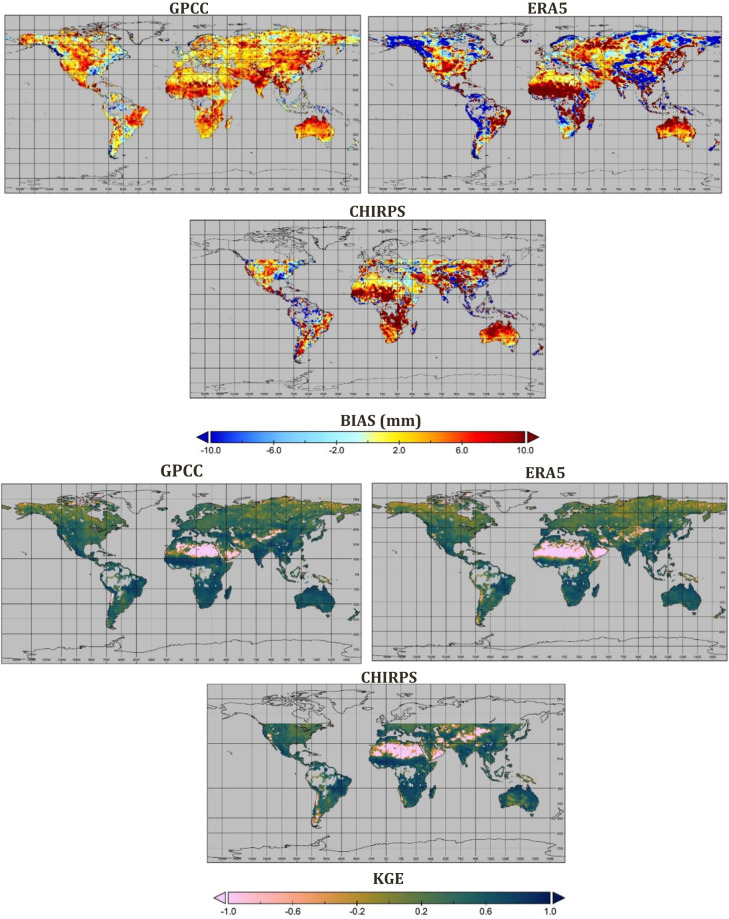
Spatial evaluation of SM2RAIN-Climate (product #1 in Table 2) dataset against GPCC, ERA5, and CHIRPS in terms of bias (BIAS) and and Kling-Gupta Efficiencies (KGE).

Figures 2, 3 show the performance of the SM2RAIN-Climate dataset against the three different global rainfall products, including GPCC, ERA5, and CHIRPS, for the period 1998–2021 at monthly timescale and 1° spatial resolution. The results indicated that SM2RAIN-Climate has a good agreement with other rainfall products. The spatial average of CC values of SM2RAIN-Climate is 0.83, 0.80, and 0.82 against the GPCC, ERA5, and CHIRPS, respectively. Based on CC and KGE values, SM2RAIN-Climate agrees well with other products over Australia, India, central and South of Africa, and the eastern part of South America. Performances are lower across the north of Africa (Sahara Desert) and the eastern part of north America, likely due to the lower temporal variability of rainfall. Moreover, due to the low quality of SM products over high latitudes (>60°), the skill performance of SM2RAIN-Climate is lower over these regions. Assessment results indicate that the RMSE value is higher over the south and eastern part of Asia than in other parts of the world. The average GPCC, ERA5, and CHIRPS BIAS values are 3.16, 1.04, and 3.17 mm, respectively. In spite of the lowest value of BIAS in ERA5 among the products, the BIAS value of this product over southeastern Asia is higher than other products, where the SM2RAIN-Climate underestimates the rainfall.

**Fig. 4:**
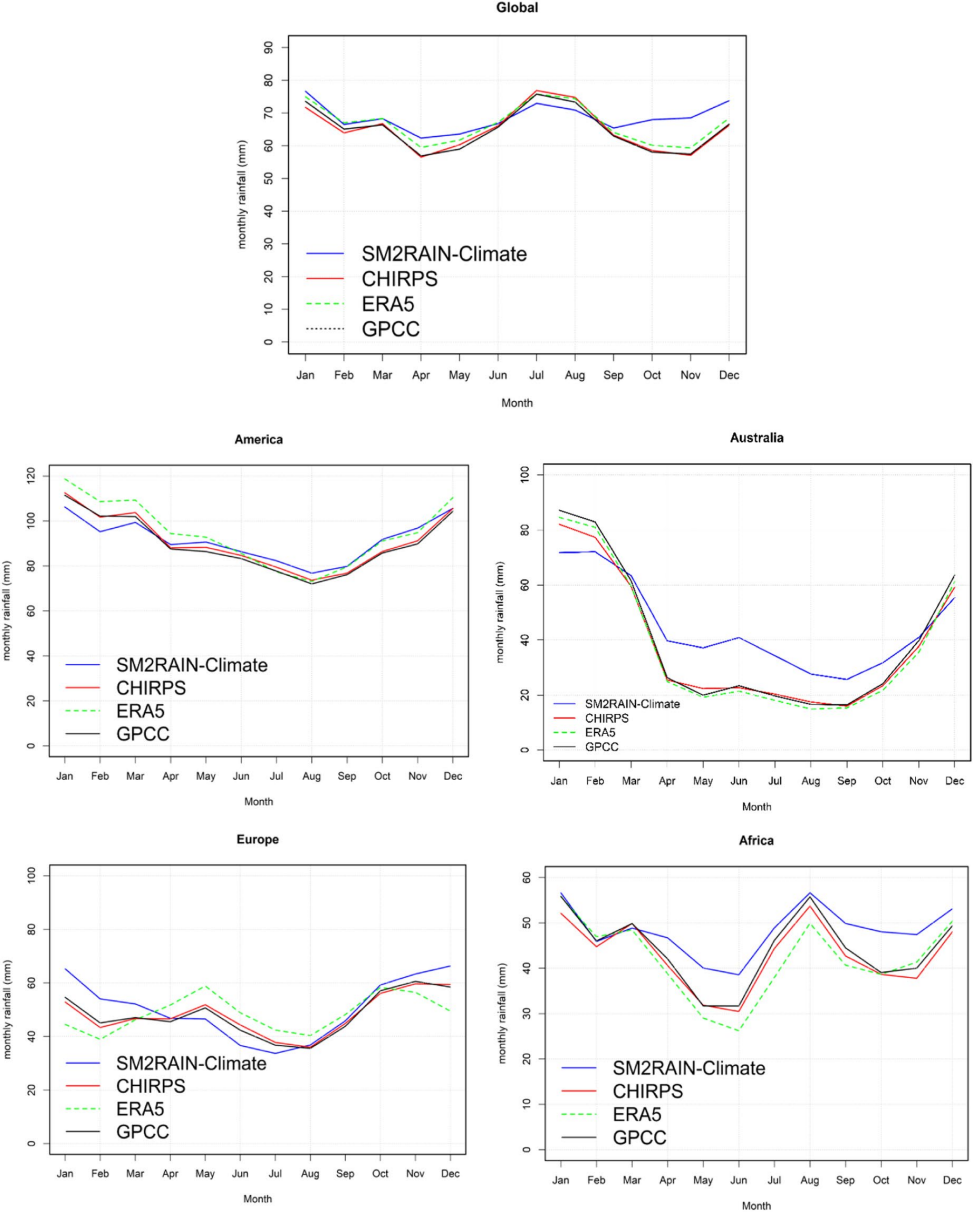
Global and regional scale seasonality (Note: America represents both South and North America).

The seasonality analysis across the globe is depicted in Fig. 4, highlighting the patterns of rainfall estimation for SM2RAIN-Climate and other rainfall products. The findings reveal that the SM2RAIN-Climate dataset estimates a higher amount of rainfall compared to the other products, particularly during the months of October, November, and December.

**Fig. 5:**
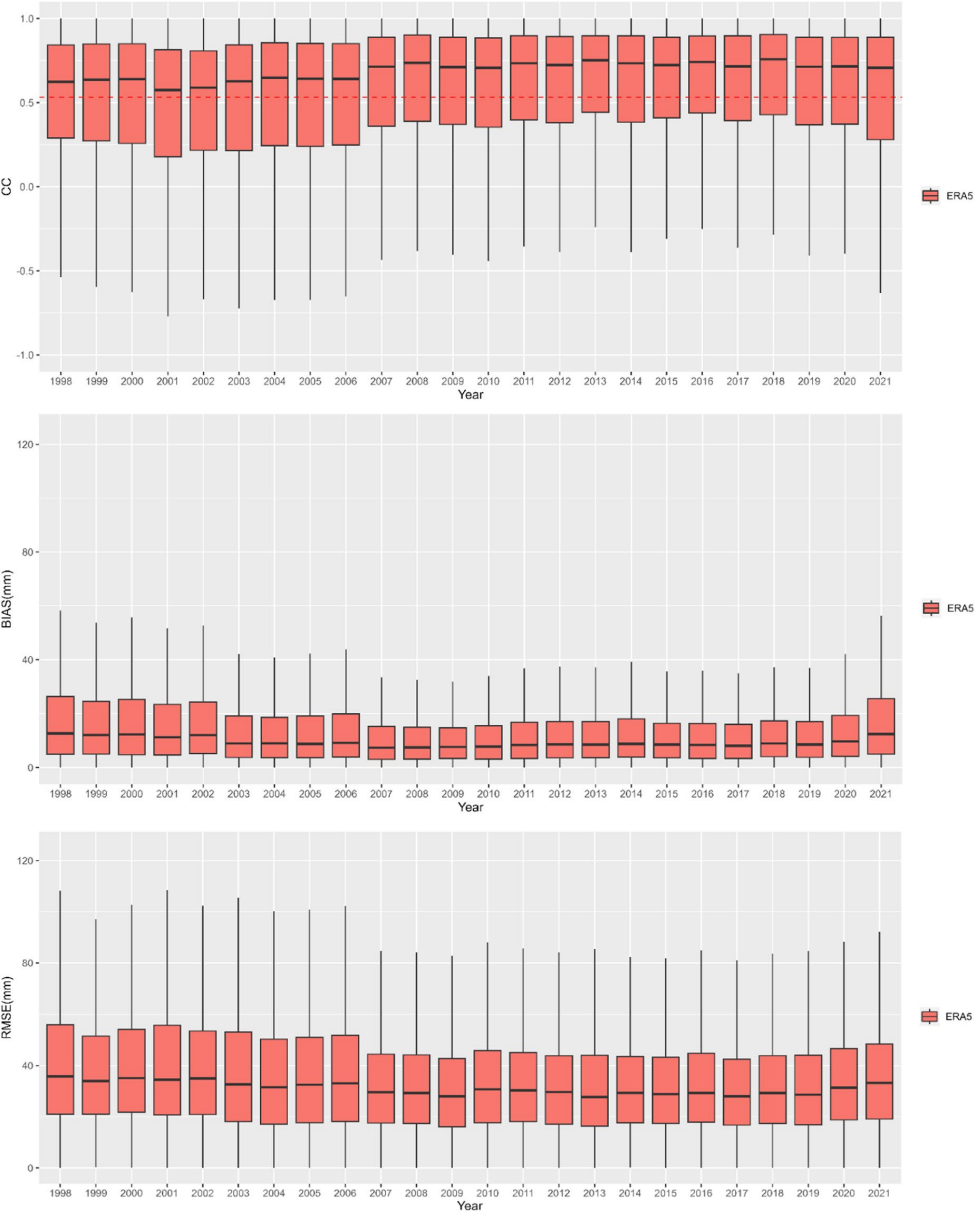
Annual boxplots of correlation coefficient (CC), root mean square error (RMSE), and bias (BIAS) value between the SM2RAIN-Climate dataset and ERA5. The red dashed line in CC figure represents the 95% significant confidence level.

Figure 5 shows the comparison results of the SM2RAIN-Climate dataset and ERA5 based on CC, RMSE, and BIAS over time, i.e., year by year. The results indicate that the performance skill is increasing over time, especially after 2007, in which the CC (RMSE and BIAS) values increase (decrease). This result should be attributed to the increase in the temporal coverage of ESA CCI SM data due to the start of the ASCAT operations.

### Regional scale assessment

**Fig. 6:**
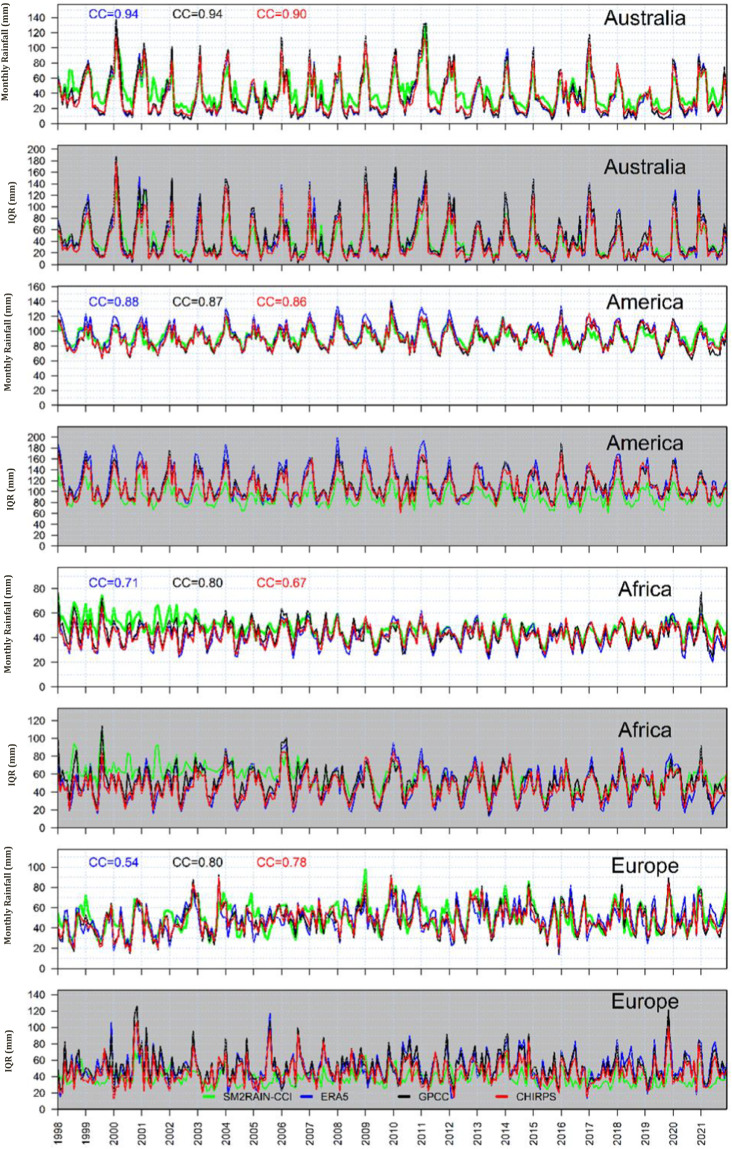
Monthly time series of four products, including SM2RAIN-Climate (green line), ERA5 (blue line), GPCC (black line), and CHIRPS (red line) spatially averaged over Australia, Africa, America, and Europe. CC represents the correlation coefficient value between spatially averaged SM2RAIN-Climate and other products.

For further investigation of the potential of SM2RAIN-Climate in the estimation of rainfall, three macroregions, including Europe, Africa, America (South and North America) and Australia, are selected. Figure 6 indicates the time series of mean monthly rainfall of four rainfall products, including SM2RAIN-Climate, GPCC, ERA5, and CHIRPS, spatially averaged over the selected regions from 1998 to 2021. In addition to the mean values, the interquartile range (IQR) time series are also calculated to provide information on the variability of rainfall within each month. The IQR reflects the range between the 25th and 75th percentiles, representing the middle 50% of the data distribution. Over Australia, the mean monthly SM2RAIN-Climate rainfall shows very good agreement with other products during the austral summer months and an overestimation in the austral winter months. Despite these differences in mean values, IQR values of SM2RAIN-Climate exhibit a remarkable consistency with the other products. This consistency in IQR values indicates that the patterns and distribution of rainfall remain relatively consistent across the different products, despite the variations in average rainfall amounts over Australia. In contrast to the situation over Australia, over America, the mean monthly SM2RAIN-Climate rainfall exhibits a relatively similar pattern compared to other rainfall products. However, there are differences in the IQR values of SM2RAIN-Climate compared to the other products. This suggests that while the average rainfall amounts are consistent among the different products, the variability and distribution of rainfall show some variations. The mean monthly rainfall and IQR value time series over Africa shows good agreement between these products except before 2007 when the SM2RAIN-Climate estimated more rainfall than other products. Additionally, the overestimation observed before 2004 can be attributed to the unavailability of active microwave data in that region prior to 2004. Overall the rainfall products have a good agreement over Europe, even though in some years there are discrepancies among products. Also, a lower IQR of SM2RAIN-Climate than other product indicates indicating less variability of monthly rainfall.

The seasonality analysis, presented in Fig. 3 for various regions, reveals consistent patterns among the products over Africa and America, indicating a convergence in capturing the seasonal variability of rainfall. Conversely, in Australia, SM2RAIN-Climate consistently exhibits higher rainfall estimates compared to other products throughout most months. Over Europe, disparities emerge between the rainfall estimates of the different products, highlighting variations in capturing the seasonal precipitation patterns within this region.

**Fig. 7:**
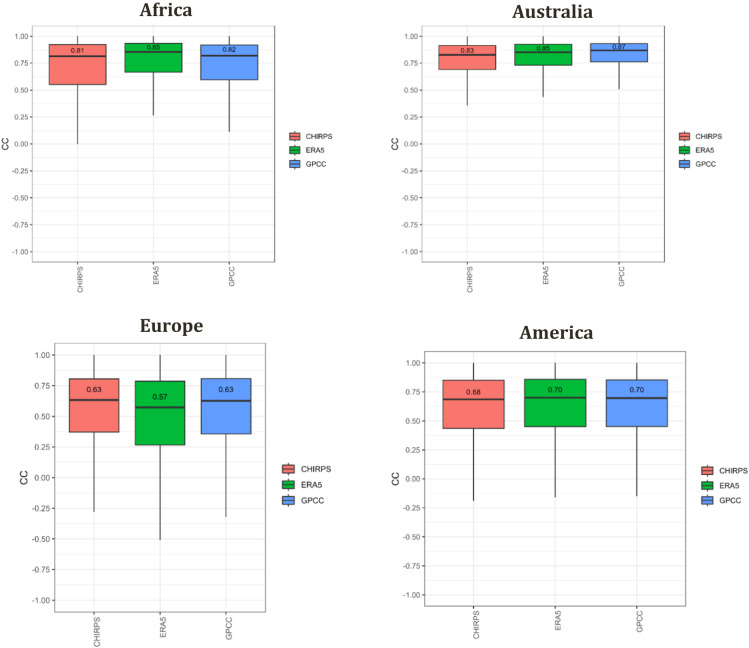
Boxplot of correlation coefficient (CC) of the SM2RAIN-Climate against three rainfall products, including CHIRPS (red box), ERA5 (green box), and GPCC (blue box) over Australia, Africa, America, and Europe.

Figure 7 shows boxplots of CC values between SM2RAIN-Climate and GPCC, ERA5, and CHIRPS. According to this result, SM2RAIN-Climate performs well in these four regions, where the median CC value is above 0.6, 0.8, 0.7 and 0.8 over Europe, Africa, America and Australia, respectively. The CC value in Australia is generally higher than in Africa and Europe. Although GPCC is used in the calibration of SM2RAIN-Climate, ERA5 has the best agreement with SM2RAIN-Climate over Africa, likely due to the poor quality of the gauge-based GPCC product in Africa.

### Trend analysis

**Fig. 8:**
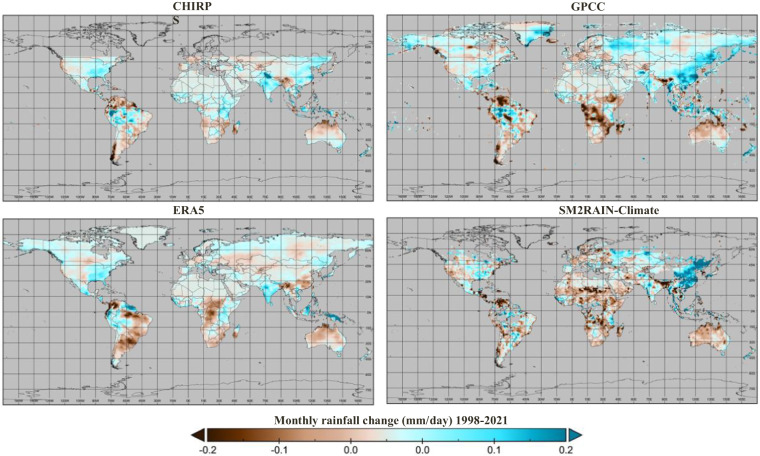
Monthly changes [mm/day] of rainfall in the period 1998–2021 with SM2RAIN-Climate, GPCC, ERA5, and CHIRPS datasets.

One of the applications of long-term rainfall products is in climate studies, e.g., trend analysis. In this section, the potential of the SM2RAIN-Climate dataset in capturing the rainfall trend is investigated and compared with the trend analysis of GPCC, ERA5, and CHIRPS. Figure 8 shows the monthly rainfall change in the different rainfall products during the period 1998–2021, calculated based on the slope of the fitted trend line, which represents the rate of change in monthly rainfall over time. The pattern of the rainfall change in all rainfall products is similar in most of the regions, such as Australia, the United States of America (USA), south and central Africa, and the northern part of South America. Also, in some regions, e.g., the eastern part of the USA, although the performances in terms of CC are not so high, the trend pattern of the datasets is very similar. In eastern Asia, the SM2RAIN-Climate trend pattern is similar to GPCC and CHIRPS but the opposite of ERA5. In this region and in others with some discrepancies, it would be highly interesting to perform detailed studies to assess the rainfall trend and the reasons for these differences.

**Fig. 9:**
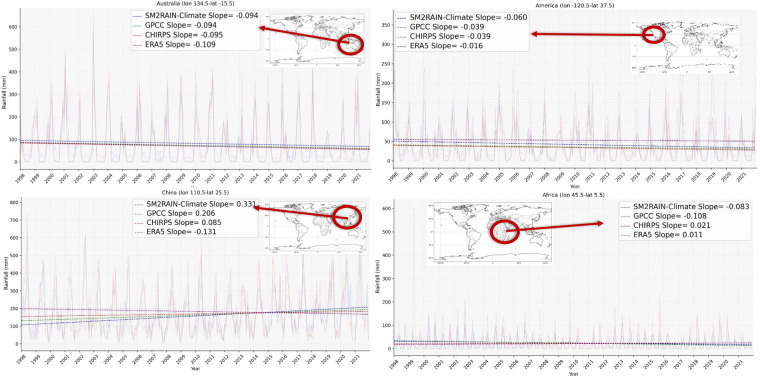
Monthly time series, trend line of rainfall change (slope in mm/day) for SM2RAIN-Climate (blue), GPCC (green), ERA5(red), and CHIRPS (purple) datasets over four specific geographic coordinate (longitude (lon) and latitude (lat)).

To investigate regional monthly rainfall trends, four specific pixels were selected for analysis. Figure 9 presents the time series of these pixels, showcasing the trend lines superimposed on the monthly rainfall data. The case study reveals both similarities and differences among the rainfall products across various regions. In the eastern part of Africa, for example, the GPCC and SM2RAIN-Climate products exhibit a declining trend, while ERA5 and CHIRPS display an opposing trend of increasing rainfall. This disparity emphasizes the significance of considering multiple datasets when assessing regional rainfall patterns. Likewise, over China, all products except ERA5 demonstrate a decreasing trend in rainfall. The inclusion of these regional time series analyses provides valuable insights into the local-scale variations in rainfall trends and further enhances the comprehensive understanding of the performance and reliability of the different rainfall products.

Scientists and stakeholders can enhance the comprehensive analysis of precipitation across various applications by utilizing a combination of rainfall products enriched with diverse informational resources. Indeed, it is worth noting that individual rainfall products can yield distinct results when compared to each other. For instance, when conducting trend analysis, the ERA5 rainfall product over East Asia and CHIRPS over East Africa display different trends compared to other datasets, which might be attributed to differences between the data sources and methodologies used for rainfall estimation.

One of the important features of the SM2RAIN-Climate dataset is the full independence from other rainfall products based on the different approaches of rainfall estimation. In this study, we show the potential of SM2RAIN-Climate rainfall estimation in many regions of the world. While we are also conscious of the SM2RAIN-Climate limitations (we expect high errors over mountainous, heavily vegetated, and frozen or snow-covered area^24^), we believe that the SM2RAIN-Climate dataset will provide additional and independent information for rainfall application and analysis.

## Data Availability

SM2RAIN algorithm code is available in python, R, and Matlab on GitHub (https://github.com/IRPIhydrology/sm2rain).

## References

[1] Zemp, M. *et al*. The status of the global climate observing system 2021: The GCOS status report. *Global Climate Observing System GCOS*. (2021).

[2] Dorigo W (2021). Closing the water cycle from observations across scales: Where do we stand?. Bulletin of the American Meteorological Society.

[3] Gebrechorkos SH, Hülsmann S, Bernhofer C (2019). Long-term trends in rainfall and temperature using high-resolution climate datasets in East Africa. Scientific reports.

[4] Pyarali K, Peng J, Disse M, Tuo Y (2022). Development and application of high resolution SPEI drought dataset for Central Asia. Scientific data.

[5] Kidd C (2017). So, how much of the Earth’s surface is covered by rain gauges?. Bulletin of the American Meteorological Society.

[6] Sun Q (2018). A review of global precipitation data sets: Data sources, estimation, and intercomparisons. Reviews of Geophysics.

[7] Massari C, Crow W, Brocca L (2017). An assessment of the performance of global rainfall estimates without ground-based observations. Hydrology and earth system sciences.

[8] Menne MJ, Durre I, Vose RS, Gleason BE, Houston TG (2012). An overview of the global historical climatology network-daily database. Journal of atmospheric and oceanic technology.

[9] Met Office (2003). NCAS British Atmospheric Data Centre..

[10] Harris I, Osborn TJ, Jones P, Lister D (2020). Version 4 of the CRU TS monthly high-resolution gridded multivariate climate dataset. Scientific data.

[11] Du J (2011). Earth Observing Laboratory.

[12] Zhang J (2016). Multi-Radar Multi-Sensor (MRMS) quantitative precipitation estimation: Initial operating capabilities. Bulletin of the American Meteorological Society.

[13] Met Office (2003). NCAS British Atmospheric Data Centre.

[14] Rienecker MM (2011). MERRA: NASA’s modern-era retrospective analysis for research and applications. Journal of climate.

[15] Ebita A (2011). The Japanese 55-year reanalysis “JRA-55”: an interim report. Sola.

[16] Gruber A, Scanlon T, van der Schalie R, Wagner W, Dorigo W (2019). Evolution of the ESA CCI Soil Moisture climate data records and their underlying merging methodology. Earth System Science Data.

[17] Brocca L (2014). Soil as a natural rain gauge: Estimating global rainfall from satellite soil moisture data. Journal of Geophysical Research: Atmospheres.

[18] Huffman, G. J., Bolvin, D. T., Nelkin, E. J. & Tan, J. Integrated Multi-satellitE Retrievals for GPM (IMERG) technical documentation. *Nasa/Gsfc Code*, **612****(****47****)** (2019).

[19] Beck HE (2019). MSWEP V2 global 3-hourly 0.1 precipitation: methodology and quantitative assessment. Bulletin of the American Meteorological Society.

[20] Roca R (2019). FROGS: a daily 1 × 1 gridded precipitation database of rain gauge, satellite and reanalysis products. *Earth System Science*. Data.

[21] Petković V (2023). Can We Estimate the Uncertainty Level of Satellite Long-Term Precipitation Records?. Journal of Applied Meteorology and Climatology.

[22] Oliveira RAJ, Roca R, Finkensieper S, Cloché S, Schröder M (2022). Evaluating the impact of a time-evolving constellation on multi-platform satellite based daily precipitation estimates. Atmospheric Research.

[23] Levizzani, V. *et al*. *Satellite precipitation measurement*. (Springer, 2020).

[24] Brocca L (2019). SM2RAIN–ASCAT (2007–2018): global daily satellite rainfall data from ASCAT soil moisture observations. Earth System Science Data.

[25] Koster RD, Liu Q, Reichle RH, Huffman GJ (2021). Improved Estimates of Pentad Precipitation Through the Merging of Independent Precipitation Data Sets. Water Resources Research.

[26] Iqbal Z (2022). Bias correction method of high-resolution satellite-based precipitation product for Peninsular Malaysia. Theoretical and Applied Climatology.

[27] Mosaffa H, Shirvani A, Khalili D, Nguyen P, Sorooshian S (2020). Post and near real-time satellite precipitation products skill over Karkheh River Basin in Iran. International Journal of Remote Sensing.

[28] Lai Y (2022). Rainfall estimation from surface soil moisture using SM2RAIN in cold mountainous areas. Journal of Hydrology.

[29] Chua Z-W, Kuleshov Y, Watkins AB, Choy S, Sun C (2022). A Comparison of Various Correction and Blending Techniques for Creating an Improved Satellite-Gauge Rainfall Dataset over Australia. Remote Sensing.

[30] Chen C (2022). Triple collocation-based error estimation and data fusion of global gridded precipitation products over the Yangtze River basin. Journal of Hydrology.

[31] Moges DM, Kmoch A, Uuemaa E (2022). Application of satellite and reanalysis precipitation products for hydrological modeling in the data-scarce Porijõgi catchment, Estonia. Journal of Hydrology: Regional Studies.

[32] Montzka, C., Bayat, B., Tewes, A., Mengen, D. & Vereecken, H. Sentinel-2 Analysis of Spruce Crown Transparency Levels and Their Environmental Drivers After Summer Drought in the Northern Eifel (Germany). *Frontiers in Forests and Global Change*, **86** (2021).

[33] Islam, Z. Soil loss assessment by RUSLE in the cloud-based platform (GEE) in Nigeria. *Modeling Earth Systems and Environment*, 1–13 (2022).

[34] Hengl T (2021). African soil properties and nutrients mapped at 30 m spatial resolution using two-scale ensemble machine learning. Scientific Reports.

[35] Paredes-Trejo F, Barbosa H, dos Santos CA (2019). Evaluation of the performance of SM2RAIN-derived rainfall products over Brazil. Remote Sensing.

[36] Satgé F (2021). Reliability of SM2RAIN precipitation datasets in comparison to gauge observations and hydrological modelling over arid regions. International Journal of Climatology.

[37] Cao D, Li H, Hou E, Song S, Lai C (2022). Assessment and Hydrological Validation of Merged Near-Real-Time Satellite Precipitation Estimates Based on the Gauge-Free Triple Collocation Approach. Remote Sensing.

[38] Abera W, Formetta G, Brocca L, Rigon R (2017). Modeling the water budget of the Upper Blue Nile basin using the JGrass-NewAge model system and satellite data. Hydrology and Earth System Sciences.

[39] Fan X (2021). Rapidly evolving controls of landslides after a strong earthquake and implications for hazard assessments. Geophysical Research Letters.

[40] Thaler S (2018). Effects of different spatial precipitation input data on crop model outputs under a Central European climate. Atmosphere.

[41] Brocca L (2016). Rainfall estimation by inverting SMOS soil moisture estimates: A comparison of different methods over Australia. Journal of Geophysical Research: Atmospheres.

[42] Tarpanelli A (2017). Exploiting a constellation of satellite soil moisture sensors for accurate rainfall estimation. Advances in Water Resources.

[43] Filippucci P (2022). High-resolution (1 km) satellite rainfall estimation from SM2RAIN applied to Sentinel-1: Po River basin as a case study. Hydrology and Earth System Sciences.

[44] Ciabatta L (2018). SM2RAIN-CCI: A new global long-term rainfall data set derived from ESA CCI soil moisture. Earth System Science Data.

[45] Koster RD, Brocca L, Crow WT, Burgin MS, De Lannoy GJ (2016). Precipitation estimation using L‐band and C‐band soil moisture retrievals. Water Resources Research.

[46] Schneider, U., Fuchs, T., Meyer-Christoffer, A. & Rudolf, B. Global precipitation analysis products of the GPCC. *Global Precipitation Climatology Centre (GPCC), DWD, Internet Publikation* **112** (2008).

[47] Muñoz-Sabater J (2021). ERA5-Land: A state-of-the-art global reanalysis dataset for land applications. Earth System Science Data.

[48] Funk C (2015). The climate hazards infrared precipitation with stations—a new environmental record for monitoring extremes. Scientific data.

[49] Dorigo W (2015). Evaluation of the ESA CCI soil moisture product using ground-based observations. Remote Sensing of Environment.

[50] Brocca, L. *et al*. Rainfall estimation from *in situ* soil moisture observations at several sites in Europe: an evaluation of the SM2RAIN algorithm. *Journal of Hydrology and Hydromechanics*, **205** (2015).

[51] Famiglietti J, Wood EF (1994). Multiscale modeling of spatially variable water and energy balance processes. Water Resources Research.

[52] Kim S (2015). A framework for combining multiple soil moisture retrievals based on maximizing temporal correlation. Geophysical Research Letters.

[53] Mosaffa H, Filippucci P, Massari C, Ciabatta L, Brocca L (2022). Zenodo.

